# Cerebral blood flow quantification in the rat: a direct comparison of arterial spin labeling MRI with radioactive microsphere PET

**DOI:** 10.1186/2191-219X-2-47

**Published:** 2012-09-15

**Authors:** Agnieszka Boś, Ralf Bergmann, Klaus Strobel, Frank Hofheinz, Jörg Steinbach, Jörg van den Hoff

**Affiliations:** 1 PET Centre, Institute of Radiopharmacy, Helmholtz-Zentrum Dresden-Rossendorf; 2 Department of Clinical Radiology, University Hospital Münster

**Keywords:** Arterial spin labeling, Cerebral blood flow, Positron emission tomography, Kinetic modeling, Blood brain barrier

## Abstract

**Background:**

Arterial spin labeling magnetic resonance imaging (ASL-MRI) has been recognised as a valuable method for non-invasive assessment of cerebral blood flow but validation studies regarding quantification accuracy by comparison against an accepted gold standard are scarce, especially in small animals. We have conducted the present study with the aim of comparing ASL flow-sensitive alternating inversion recovery (FAIR)-derived unidirectional water uptake (*K*_1_) and ^68^Ga/^64^Cu microsphere (MS)-derived blood flow (*f*) in the rat brain.

**Methods:**

In 15 animals, *K*_1_and *f* were determined successively in dedicated small animal positron emission tomography and MR scanners. The Renkin-Crone model modified by a scaling factor was used for the quantification of *f* and *K*_1_.

**Results:**

Below about 1 mL/min/mL, we obtain an approximately linear relationship between *f* and *K*_1_. At higher flow values, the limited permeability of water at the blood brain barrier becomes apparent. Within the accessed dynamic flow range (0.2 to 1.9 mL/min/mL), the data are adequately described by the Renkin-Crone model yielding a permeability surface area product of (1.53±0.46) mL/min/mL.

**Conclusion:**

The ASL-FAIR technique is suitable for absolute blood flow quantification in the rat brain when using a one-compartment model including a suitable extraction correction for data evaluation.

**Trial registration:**

24-9168.21-4/2004-1 (registered in Freistadt Sachsen, Landesdirektion Dresden)

## Background

Arterial spin labeling magnetic resonance imaging (ASL-MRI) has been widely recognised as a valuable method for non-invasive assessment of regional cerebral blood flow (rCBF) [1-4]. Similar to ^15^O-water positron emission tomography (PET), the method is based on the assumption of free (or at least very high) diffusibility of water between blood and tissue. In contrast to PET, however, the relevant time scales for the measurement are much shorter (of the order of a few seconds) due to the rapid free decay of the longitudinal magnetisation of magnetically tagged water. At the same time, the temporal resolution is much higher than in PET. For these reasons, it is not clear to what extent the simple Kety-Schmidt model [5,6] suffices for reliable perfusion quantitation with ASL. Several refinements and modifications of the basic model have been proposed in the literature [7-9].

Regarding human imaging, ASL nowadays is in general applied in a qualitative way only using regional image contrast as a relative measure of perfusion differences. On the other hand, a number of studies have compared ASL-MRI to other methods, notably ^15^O-water PET [10-12] and found reasonable or even good quantitative concordance between MR- and PET-derived rCBF. The principal feasibility of quantitative ASL in humans might become especially interesting in view of the recent advent of combined PET/MRI systems, offering the perspective of combined functional imaging utilising both modalities.

With respect to small-animal imaging, however, there still seems to exist a marked deficit of validation studies demonstrating the quantitative accuracy of ASL, and reliable data are scarce [7,13,14]. Moreover, there are indications that water diffusibility across the blood brain barrier (BBB) is severely reduced in the rat brain [15,16] with corresponding implications for blood flow quantitation with ASL.

We, therefore, consider the validity of ASL for rCBF quantitation in small laboratory animals, which still is not proven conclusively. We have thus conducted the present study with the aim of comparing the flow-sensitive alternating inversion recovery (FAIR) technique in the rat brain with microsphere-derived rCBF.

Radioactively labeled microspheres are an accepted gold standard for perfusion quantification due to the well-understood behaviour of microspheres in the vascular bed and the inherent ease of quantitation of radioactive tracer amounts. In the current investigations, we used dedicated small animal PET imaging for in-vivo quantification of the microsphere experiments. In order to double-check the PET results, the microspheres were labeled not only with PET isotopes but also with a fluorescent dye. This allowed two independent measurements of the number of microspheres trapped in the capillary bed, namely in-vivo with PET and ex-vivo via direct counting of microspheres in the brain sections.

## Methods

### Microsphere labeling

Human serum albumin microspheres (HSAMS) (20 *μ*m, 1 mg ∼ 160 000, ROTOP Pharmaka AG, Germany) were mixed in proportion 1:70 with p-SCN-Bn-NOTA. Afterwards, chelator-bound microspheres (NOTA-HSAMS) were labeled with X-SIGHT Large Stokes Shift Dye (XS670) (Carestream Health Deutschland GmbH, Stuttgart, Germany) under reduced light conditions according to producer specification. Both, chelator preparation and labeling procedure, were performed under strict exclusion of metals (plastic vessels and coated spatulas).

Gallium-68 (half-life, 68 min) was eluted as [^68^Ga]GaCl_3_ with 1 M HCl from a ^68^Ge/^68^Ga-generator (iThemba Labs, Republic of South Africa) and concentrated via an ion exchange column to a volume of 300 *μ*L. NOTA-XS670-HSAMS were washed (centrifugation 14,800 rpm, 10 s) with 1 mL of water and 1 mL 2 M NH_4_OAc. The pH of the [^68^Ga]GaCl_3_ solution was set up to 4.2 to 4.4 using 2 M NH_4_OAc and added to the washed microspheres. The labeling was carried out at 37°C for 20 min in thermomixer. Labeling yields were determined by measuring of the microspheres and comparing the activity of the supernatant after centrifugation of three times washed [^68^Ga]Ga-NOTA-XS650-HSAMS using 0.1% Tween 80 (Carl Roth GmbH + Co. KG, Karlsruhe, Germany) in E153 (Serumwerk Bernburg, Germany). This method resulted in reproducible high yields of ≥95% microsphere-associated activity. The [^68^Ga]Ga-NOTA-XS670-HSAMS were washed directly before application, reaching a radiochemical purity higher than 99%.

Copper-64 (half-life, 12.7 h; produced in cyclotron ‘Cyclone 18/9’ of the HZDR according to the procedure reported previously [17,18]) as aqueous solution of ^64^Cu]CuCl_2_ was adjusted with 2 M NH_4_OAc to pH 5.5. The NOTA-XS670-HSAMS were washed with 2 M NH_4_OAc. Then, the ^64^Cu]CuCl_2_ solution was added to the microspheres and incubated at 37°C for 20 min in the termomixer. Labeling yields were determined in the same way as for ^68^Ga.

Ten animals were investigated with ^68^Ga-labeled microspheres, and eight animals with ^64^Cu-labeled microspheres. The radionuclide was chosen according to availability, where ^64^Cu was preferred because of its longer half-life. The chosen radionuclide determines the order of MR and PET measurements, but the altered time of the PET measurement does not affect the derived flow values (which are those present at the time of microsphere injection, see below).

### Animal preparation

#### Animal husbandry

The local animal research committee approved the animal facilities and the experiments according to institutional guidelines and the German animal welfare regulations (reference number 24-9168.21-4/2004-1). The experimental procedure conforms to the European Convention for the Protection of Vertebrate Animals used for Experimental and other Scientific Purposes (ETS No. 123), to the Deutsches Tierschutzgesetz, and to the Guide for the Care and Use of Laboratory Animals published by the US National Institutes of Health (DHEW Publication No. (NIH) 82-23, Revised 1996, Office of Science and Health Reports, DRR/NIH, Bethesda, MD 20205).

#### Surgery

Male Wistar rats (Wistar Unilever, HsdCpb:WU, Harlan Winkelmann, Borchen, Germany) weighting 379 ± 65 g (mean ± SD, *n* = 18) were anaesthetised with desflurane (approximately 7% in 0.6 L/min air and 0.4 L/min O_2_). The guide value for breathing frequency was 65 breaths/min. Animals were put in the supine position and placed on a heating pad to maintain body temperature. The spontaneously breathing rats were treated with 100 units/kg heparin (Heparin-Natrium 25000-ratiopharm, Ratiopharm, Germany) by subcutaneous injection to prevent blood clotting on intravascular catheter. The Lignocaine 1% (Xylocitin loc, Mibe, Germany) was injected into the right groin. A patch of skin and fat tissue next to the groin was removed and blood vessels were separated from surrounding tissue. The distal end of the artery was ligated with suture. A heparin filled catheter (0.8 mm Umbilical Vessel Catheter, Tyco Healthcare, Ireland) was connected to the syringe and inserted through an incision in the clamped artery. Finally, the distal ligation was tied to the cannula. Then, the syringe for withdrawing the reference arterial blood samples was attached to a Standard Infuse/Withdraw PHD 22/2000 Syringe Pump (Harvard Apparatus, Massachusetts, USA).

The installation of the cardiac ventricle catheter was performed in several steps previously described. The catheter was afterwards connected to the blood pressure monitoring device, and the cannula was advanced towards the heart. The catheter insertion proceeded until the blood pressure signal vanished. The catheter was then retracted 2 mm, and the end of caudate ligation was tied around by a rostal knot. The blood pressure device was then replaced by the syringe containing the microspheres. The syringe for reference arterial blood samples was attached to a Standard Infuse/Withdraw PHD 44 Syringe Pump (Harvard Apparatus, USA). The position of the cannula in the left ventricle was confirmed by computer tomography (CT) (Skyscan 1178, Skyscan, Belgium). The syringe with the microspheres (approximately 160,000 microspheres in 1 mL E-153) was sonicated at room temperature and quickly connected to the ventricular catheter. The syringe pump for extraction of the arterial reference blood sample was started with a speed of 0.5 mL/min and continued over 2 min. The microspheres were injected 0.5 min after starting the reference blood sampling with a rate of 1 mL/min.

### Experimental protocol

**Figure 1:**
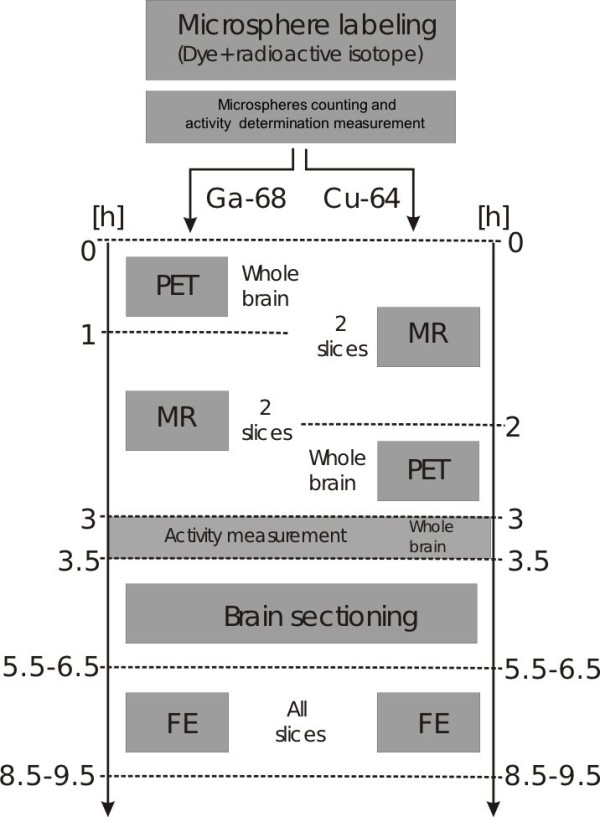
Work flow of the performed experiments.

The experimental protocol is summarised in Figure 1.

#### Microsphere injection

_64_Cu-labeled microspheres (48 ± 27 MBq; 148,755 ± 49,103 microspheres; 8 animals) and _68_Ga-labeled microspheres (190 ± 79 MBq; 149,068 ± 34,530 microspheres; 10 animals) were injected resulting in 4.1 ± 1.7 MBq _64_Cu- and 13.2 ± 9.6 MBq _68_Ga activity, respectively, in the PET field of view. After injection of the microspheres, the animal was placed on a dedicated heated animal bed allowing both MRI and PET imaging without animal repositioning. After stabilisation of the animal over 5 min, the MRI or PET investigations were performed. Animal preparation and tomographic imaging procedure took about 3 h (MRI, 120 min; PET, 60 min). The body temperature and heart and breath rates of the animals were monitored by a MR-compatible small animal monitoring and gating system (SAM system, model 1025/1025L, SA Instruments Inc, USA) during the MRI and PET examinations. The temperature of the animals was stabilised by adjusting the temperature of the animal bed. Breathing and heart beat rates were stabilised by controlling the amount of inhaled anaesthetic gas.

PET imaging had to be performed prior to MR imaging for the animals injected with _68_Ga-labeled microspheres due to the limited radioactive half-life of this isotope. MR imaging was performed first for animals injected with _64_Cu-labeled microspheres. After the completion of all measurements (PET, MRI), the animal was killed, and the brain was extracted and weighed. Radioactivity was measured in a dose calibrator cross-calibrated to the PET scanner (Isomed 2000, Med Nuklear Medizintechnik, Germany). The dose calibrator was also used for the radioactivity measurements of the microsphere standard and the reference blood sample. Finally, the brain was quickly frozen by immersion in isopentane/dry ice solution and temporarily stored (less than 24 h) until it was sliced into 40-*μ*m thick sections in a cryostat microtome (Leica CM1850, Leica Mikrosysteme Vertrieb GmbH Mikroskopie und Histologie, Germany). Each section was placed on a microscope slide (Carl Roth GmbH + CO. KG, Germany). Cryosectioning of the brain and fluorescence examination (FE) took around 5 to 6 h.

#### MRI examination

Measurements were performed with a 7 T small animal MRI system (BioSpec 70/30, Bruker, Germany), with the vendor provided ASL protocol using a FAIR technique with an adiabatic hyperbolic secant inversion pulse (length-bandwidth product 120) and echo planar imaging acquisition (spin echo, TE = 14.67 ms, TR = 9,000 ms, NA = 5, NR = 1). During each experiment, the rat was placed in the middle of the homogeneous field of the resonator, prone with head first, and the rat head coil (RAT-BR Q-REC (SN) 300-1H, Bruker BioSpin MRI GmbH, Germany) placed just above the brain. T_2_-weighted 2D and 3D images were acquired (turbo rapid acquisition with refocused echoes (RARE)-T_2_: TE = 11 ms, TR = 2,742 ms, NA = 3, rare factor = 8, FOV = 40×40×1 mm, matrix = 128×128×13, inter-slice distance = 1.5 mm; turboRARE-3D: TE = 9 ms, TR = 1,500 ms, rare factor = 16, FOV = 40×40×40 mm, matrix = 128×128×128). These data were used for spatial registration of the MR and PET data sets and selection of individual slices for the ASL measurements. Single-shot/multi-phase ASL measurements (inversion recovery time (TIR) = 26 ms, increment of TIR = 200 ms, number of TIR = 22) were performed in two transaxial slices intersecting the central region of the brain and the cerebellum (Bregma – 3.8±0.5 and – 9.68±0.5 mm, respectively). Time points of the T_1_-relaxation curve were collected successively for each voxel of both planes which took 33 min (2×16.5 min). The resulting relaxation data were evaluated with in-house software written in the R programming language [19], yielding parametric maps of tissue perfusion.

#### PET examination

The in-vivo distribution of radiolabeled microspheres was assessed with small animal PET (microPET P4, Siemens Medical Solutions, Germany) by acquiring a static 60 min frame and by performing ROI (region of interest) analysis in the 3D tomographic data (FOV = 76.8×76.8×7.62 mm, maximum a posteriori image reconstruction, matrix = 256×256×63) corrected for attenuation using an 10 min scan with a point source (^57^Co). A common animal bed was used in the MRI and PET examination, and repositioning of the animal on the bed was avoided which ensures good hardware-based coregistration of both investigations. Residual misalignment was corrected by manual registration between the anatomical MR and PET images with the ROVER software (ABX GmbH, Radeberg, Germany) [20] using MR as the reference image. The activity distribution was analysed in ROIs, matching those used in the ASL measurements.

#### Fluorescence examination

Selected sections obtained from cryosectioning of the brain were examined with an optical imaging system (FX Pro, Carestream Health Deutschland GmbH, Stuttgart, Germany), and the number of microspheres in each section was counted manually on screen. The relevant sections were selected by visual comparison with the anatomical MRI slices obtained during the MRI examination of the respective animal. For each MRI slice (thickness 1 mm), a range of 25 consecutive sections (thickness 0.04 mm) was selected. The accuracy of the spatial correspondence between brain sections and MRI slices was about 0.5 mm.

Reference blood samples were dried on filter paper (Carl Roth GmbH + Co. Kg, Germany) and analysed using the technique mentioned above. This enabled the determination of the number of microspheres in 1 mL blood. From the separately determined total radioactivity of the blood sample, the average activity bound to a single microsphere was then calculated.

### Data evaluation procedures

#### Data integrity check

Data integrity was first checked by comparing the total radioactivity in the brain determined in two independent ways: 

● FE: direct counting of microspheres in all brain sections

● PET: whole brain ROI

The FE-derived activities were, moreover, compared with ex-vivo measurements of the brain activity (EXT) in a dose calibrator after brain extraction. For all animals, FE and EXT correlated very well (Pearson correlation 0.99), demonstrating the validity of the FE-based evaluation.

Direct in-vivo counting of the fluorescent microspheres could be carried out in 13 out of 18 animals. The FE- and PET-derived total brain activities were then compared. Out of the 13 assessed animals, three animals were excluded where the difference between both values was larger than 25%.

For the ten animals passing this check as well as for the remaining five animals where FE could not be performed, we then checked for sufficient stability of temperature (35^°^C to 37^°^C), respiration (51 to 89 breaths/min), and heart rate (192 to 388 bpm) during all measurements. It turned out that the long duration of the two successive MR measurements of both selected slices in each animal partly led to sizable fluctuations of these physiological parameters between the MR measurements (and in comparison to the PET measurement). This affected the cerebellum slice MR measurement in five animals. These measurements were therefore excluded from further evaluation. Moreover, we had one drop out by technical failure of a single slice MR measurement in one of the animals.

Altogether, we could include data from 15 out of 18 investigated animals, excluding six out of a total of 15·2=30 slices (five due to instabilities of physiological parameters, one technical drop out). The comparison between microsphere-derived perfusion and ASL thus encompassed a total of 24 brain slices.

#### Image coregistration

For all 15 animals included in the final evaluation, MR and PET image volumes were spatially registered using the ROVER software. Perfusion quantitation was then performed separately in the PET and ASL-MR data for the two slices which had been selected for the ASL measurements. The quantitation was performed as described in the following.

#### ASL-FAIR measurements

ASL uses magnetically labeled arterial water protons for perfusion measurements in a fashion principally similar to ^15^O-H_2_O PET, although time scales, magnitude of the flow-sensitive signal, and type of labeling are quite different.

**Figure 2:**
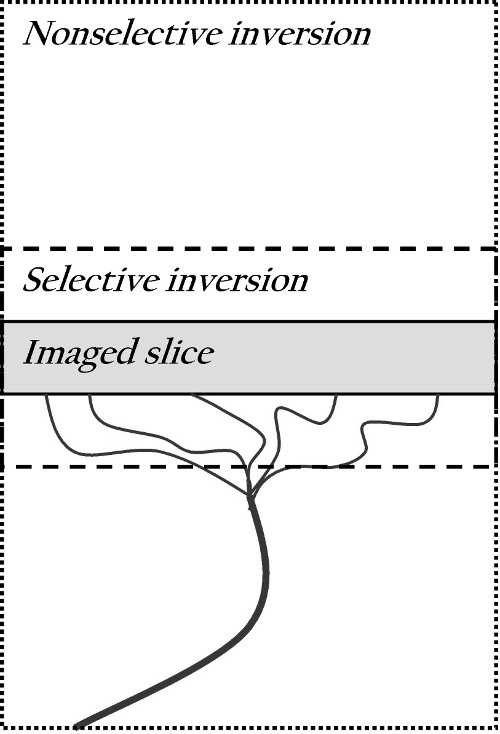
**Schematic diagram of the FAIR technique.** Blood flows up through the artery into the imaged slice. Two images are acquired: a selective inversion image, where spins are labeled with a 180° pulse in the selected plane and a nonselective image, where the whole volume is labeled with 180°.

The FAIR technique (see Figure 2) uses slice-selective inversion labeling of the targeted slice (including a small neighbourhood of ± 1 mm at a target slice thickness of 1 mm) and independent global inversion labeling which covers the whole animal (or at least a sufficiently large fraction, ensuring that no unlabeled blood enters the imaged slice during the measurement, in our case the first 4.5 s after inversion). We describe the transport kinetics of the water molecules with a one-tissue compartment model, but we account for the possibility of limited first-pass extraction across the BBB by introducing a flow-dependent uptake rate *K*_1_ and a tissue clearance rate *k*_2_which have the flow-independent ratio

(1) $$V_{d}=\frac{K_{1}}{k_{2}},$$

representing the volume of distribution of water in tissue. *K*_1_ is related to blood flow by

(2) $$K_{1}(f)=E(f)\cdot f,$$

where *E*(*f*) is a flow-dependent extraction fraction, which becomes equal to 1 if the BBB is considered perfectly permeable for water.

We use the abbreviation

(3) $$m(t)=\frac{M_{0}-M(t)}{2}$$

for (half) the difference between equilibrium magnetisation *M*_0_ and the instantaneous value *M*(*t*) at time *t* after complete inversion, i.e. *m*(0)=*M*_0_and *m*=0 at equilibrium. Using further

(4) $$\rho =\frac{1}{T1}$$

for the T_1_-related relaxation rates, we arrive at this differential equation for the ASL one-tissue compartment model [21]

(5) $$\frac{dm(t)}{dt}=K_{1}m_{a}(t)-\rho_{s}m(t)\;\;\text{with:}\;m(0)=M_{0},$$

where *m*_*a*_ is the arterial input function, *ρ*_*s*_=*k*_2_ + *ρ*_0_ is the apparent, and *ρ*_0_the true T_1_-relaxation rate in tissue. For our FAIR measurements, the input function is assumed to be equal to zero in the selective measurement and equal to

(6) $$m_{a}(t)=m_{a0}\;e^{-\rho_{a}t}=\frac{M_{0}}{V_{d}}e^{-\rho_{a}t}$$

in the global measurement, where *ρ*_*a*_is the T_1_-relaxation rate in the incoming arterial blood. We assume that the tissue-to-blood ratio of the saturation magnetisation equals the distribution volume of water.

From these equations, we obtain the tissue response curves *m*_*s*_(*t*) and *m*_*g*_(*t*) for selective and global measurement, respectively. *m*_*s*_ is mono-exponential with relaxation rate *ρ*_*s*_. *m*_*g*_is a weighted sum of two exponentials with numerically similar rate constants *ρ*_*s*_ and *ρ*_*a*_, respectively. Since the resulting curve is practically indistinguishable from a single exponential, *m*_*g*_(*t*) is adequately described by a single effective relaxation rate *ρ*_*g*_. Going back to the actual magnetisation *M*(*t*) (see Equation 3), we obtain the following operational equations modeling the measured relaxation curves:

$$\begin{array}{ll} M_{s}(t)=M_{0}\cdot \left(1-2\cdot e^{-\rho_{s}t}\right) & \;\\ M_{g}(t)=M_{0}\cdot \left(1-2\cdot e^{-\rho_{g}t}\right). & \; \end{array}$$

The three parameters *M*_0_, *ρ*_*s*_, and *ρ*_*g*_ are determined independently for each voxel with a simultaneous least squares fit of these equations to the measured selective and global relaxation curves.

Further computation leads to this relation between the experimentally accessible relaxation rates, *ρ*_*s*_ and *ρ*_*g*_, and the tissue clearance rate *k*_2_[22-24]:

(7) $$k_{2}=\frac{\rho_{a}}{\rho_{g}}\cdot (\rho_{s}-\rho_{g}).$$

from which *K*_1_follows (assuming *V*_*d*_and *ρ*_*a*_ are known):

(8) $$K_{1}=V_{d}\cdot k_{2}=V_{d}\cdot \rho_{a}\;\frac{\rho_{s}-\rho_{g}}{\rho_{g}}$$

In our data evaluation, we have used the value *T*_1*a*_=1/*ρ*_*a*_= 2.0 s [25] and different *V*_*d*_values, representative of cortex and cerebellum, for the two investigated slices, namely *V*_*d*_=0.95 mL/mL (Bregma-3.8 mm) and 0.88 mL/mL (Bregma-9.68 mm), respectively [26]. For comparison with the microsphere measurements, the resulting *K*_1_values were finally averaged over the respective slices.

#### Microsphere measurements

Blood flow quantification with microspheres requires arterial injection of suitably sized microspheres (upstream of the target organ) which are then trapped completely during the first pass through the capillary bed. Assuming uniform radioactive labeling of all microspheres, the measured level of radioactivity in a tissue sample is proportional to the number of microspheres. The relation between accumulated activity *A* [Bq] in the given sample volume and total blood flow *F* (mL/min) through the sample is given by

(9) $$A=F\cdot \int_{0}^{\infty }c_{a}(t)\text{dt},$$

where *c*_*a*_(*t*) is the time dependent concentration of microsphere-associated radioactivity in the arterial blood (Bq/mL). Plotting *c*_*a*_(*t*) over time, the integral in Equation 9 is simply identical to the area under the curve. Assuming sufficient mixing of the microspheres in the left ventricle, *c*_*a*_(*t*) is the same for all arteries, and the blood flow *F*_*t*_in a target region can be derived by comparison of the target region activity *A*_*t*_ with a ‘reference organ’ obeying, too, Equation 9. This reference is created by drawing arterial blood samples at a known fixed flow rate *F*_*r*_ and measuring the activity *A*_*r*_in the resulting reference blood sample. Then, the following relation directly follows:

$$\frac{A_{t}}{A_{r}}=\frac{F_{t}\cdot \int_{0}^{\infty }c_{a}(t)\text{dt}}{F_{r}\cdot \int_{0}^{\infty }c_{a}(t)\text{dt}}=\frac{F_{t}}{F_{r}}$$

and, thus,

(10) $$F_{t}=\frac{F_{r}}{A_{r}}\cdot A_{t}.$$

In the context of PET, it is more reasonable to use regional activity concentrations *c*_*t*_(Bq/mL), i.e. activity per unit volume of perfused tissue, which is the quantity actually measured by PET and to normalise the blood flow to unit volume accordingly. Using the symbol *f* for this normalised blood flow (mL(blood)/min/mL(tissue)), Equation 10 is replaced by

(11) $$f=\frac{F_{r}}{A_{r}}\cdot c_{t},$$

where, in our case, *f* is identical to the rCBF. As can be seen, the measurement requires to continuously draw an arterial blood sample at the fixed flow rate *F*_*r*_ and measurement of the total activity *A*_*r*_in the resulting sample. Sampling has to start before the first microspheres arrive at the sampling site and to continue until the last microspheres have passed by. The tissue radioactivity concentration *c*_*t*_ was determined in several ways for cross-validation purposes. First, *c*_*t*_was determined in vivo in the reconstructed 3D PET image volume for the relevant slices (corresponding to those for which the ASL measurements were performed) via suitable ROI definition as well as for the whole brain. In 9 out of the 15 animals which were included in the final data evaluation (see section data integrity check), we were also able to perform ex-vivo radioactivity measurements of the whole brain using a dose calibrator. Finally, the brains were analysed after sectioning by direct counting of the fluorescent microspheres (FE) and conversion of the number of microspheres to amount of activity using the known average amount of radioactivity bound to a single microsphere: 0.316 ± 0.198 kBq for ^64^Cu, and 1.248 ± 0.495 kBq for ^68^Ga. The tissue weights were converted to tissue volumes assuming a density of 1.04 g/mL [27].

#### Comparison of ASL and microsphere measurements

For comparison of the ASL-derived uptake *K*_1_and the microsphere-derived blood flow *f*, we use the Renkin-Crone model which provides this relation for the flow-dependent extraction *E* in Equation 2 [28,29]:

(12) $$E(f)=1-e^{-\frac{\text{PS}}{f}}$$

which leads to

(13) $$K_{1}(f)=f\left(1-e^{-\frac{\text{PS}}{f}}\right),$$

where *PS* is the permeability surface area product of water at the BBB. In order to account for potential bias introduced by errors in the chosen value of the constant factor *V*_*d*_·*ρ*_*a*_appearing in Equation 8, we augment this formula by an additional scaling factor *N*:

(14) $$K_{1}(f)=N\cdot f\left(1-e^{-\frac{\text{PS}}{f}}\right).$$

We have used Equations 13 (free parameter: *PS*) and 14 (free parameters: *N* and *PS*) for least squares fits of the ASL-derived *K*_1_values vs. the microsphere-derived flow values.

## Results

**Figure 3:**
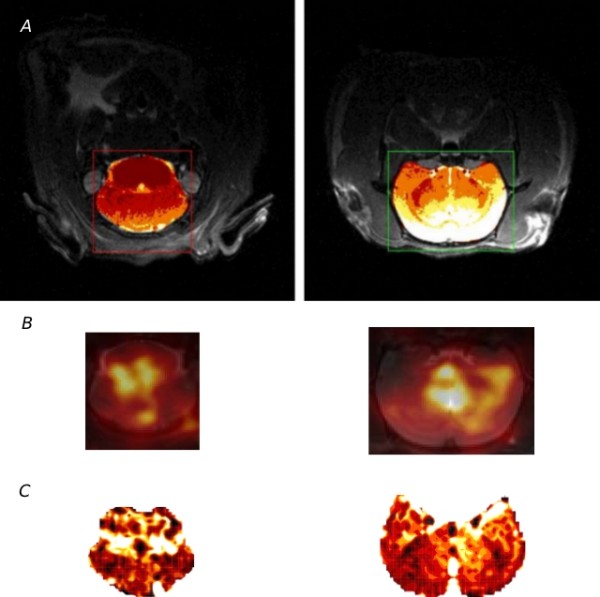
**Direct comparison of the two sections marked on MR-anatomical image on the top.** (**A**) radioactively labeled microsphere distribution measured by PET overlayed with MR-anatomical image (**B**) and ASL perfusion parametric image (**C**).

**Figure 4:**
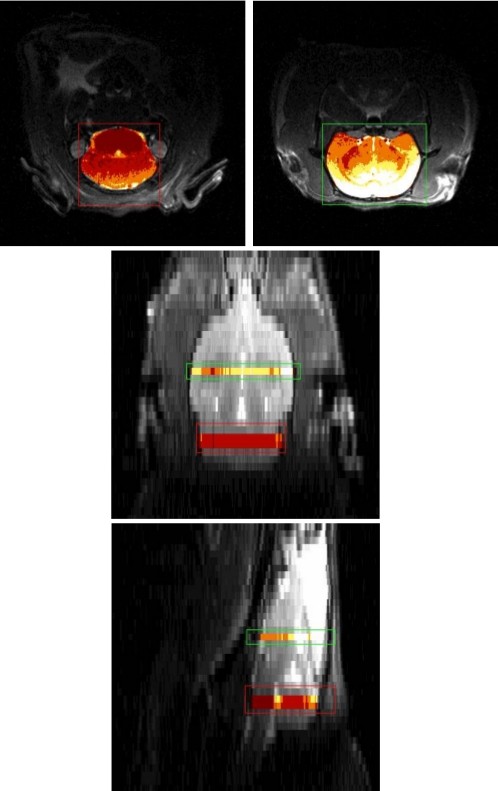
**Transaxial (top), coronal (middle), and sagittal (bottom) views of the rat brain.** The regions selected for ASL-MRI are indicated in a separate color map.

Figure 3 shows an example of the spatially coregistered MS-derived perfusion distribution (*f*) and ASL-derived unidirectional water uptake (*K*_1_) for the animal from Figure 4. Due to the rather limited statistical accuracy of these images (and the limited number of microspheres in the corresponding brain sections used for direct microsphere counting), we compared only the slice averages of these data. The transaxial slice thickness was 1 mm in MRI and 3 mm in PET. The larger slice thickness for PET is a consequence of the inferior spatial resolution of this method and does affect, to some extent, the accuracy of spatial correspondence between both data sets.

**Figure 5:**
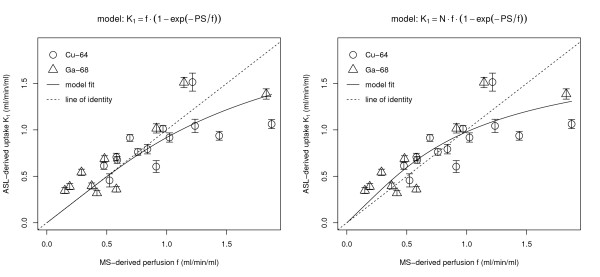
**Least squares fit of the Renkin-Crone model to the data.** Left: standard Renkin-Crone model (Equation 13). Right: Renkin-Crone model with additional scaling factor (Equation 14).

The obtained 24 pairs of slice-averaged perfusion/uptake values were further evaluated by least squares fits of the model Equations 13 and 14 to these data. The results are shown in Figure 5.

Both fits exhibit comparable overall quality, but introduction of the scaling factor *N* does lead to a slight reduction of the residual standard error (sum of squared residuals divided by degrees of freedom of the respective fit) which was 0.216 and 0.206 for standard and modified Renkin-Crone model, respectively. Especially notable is improvement at low flows where the standard fit tends to underestimate the measured data. We, therefore, adopt the fit including the scaling parameter as our final result. The resulting fitted parameters are

$$\begin{array}{lll} N & =1.25\pm 0.17\; & \;\\ \text{PS} & =(1.53\pm 0.46)\;\text{mL/min/mL}.\; & \; \end{array}$$

It is, moreover, apparent from Figure 5 that there is no systematic difference between the results obtained with _64_Cu (circles) and _68_Ga (triangles), respectively.

## Discussion

In human brain studies, the capability of ASL to provide perfusion values which are consistent with those delivered by ^15^O-H_2_O PET can be considered established [10-12,30]. The situation is less clear in small animal imaging, however. Despite the fact that there are many studies reporting on the use of ASL in pre-clinical imaging, there are only very few investigations regarding the quantitative accuracy of ASL-derived perfusion values [7,8,13,14].

We have, therefore, conducted the present study with the aim of evaluating the quantitative accuracy of one common ASL technique, namely ASL-FAIR, for brain imaging in rats by a direct comparison with radioactive microspheres which are an accepted gold standard for perfusion quantification.

Our final results are presented in Figure 5 together with fits of two related models to the data. Although there is substantial scatter, we observe a clear monotonic relation between MS-derived brain perfusion *f* and ASL-derived unidirectional water uptake *K*_1_. Each data point results from averages over spatially registered transaxial brain slices with comparable thickness (1 mm in MRI, 3 mm in PET). The points can, therefore, be considered as representing the slice averages of the respective parameter (*f* and *K*_1_). It would have been desirable to compare *f* and *K*_1_also on a regional basis within the slices. However, the limited number of microspheres present in a single slice does not allow a reliable determination of perfusion values for different slice regions. The substantial dynamic range of the MS-derived flow (and the ASL-derived *K*_1_) values (ranging from about 0.2 to 1.9 mL/min/mL) might be surprising since no special measures were taken to achieve different flow levels in the different animals. The range can be explained by observing that the investigated animal group was heterogeneous with respect to age and weight and, more importantly, that the animal preparation (notably catheterisation and anaesthesia) does necessarily influence the individual animals differently.

The resulting large variability of the resting blood flow is actually advantageous in the present investigation since it made additional intervention (pharmacological or pacing) aiming at variation of blood flow unnecessary.

The observed correlation between *f* and *K*_1_is approximately linear as can be seen by comparison with the line of identity in the plots. At high flow values, however, there is some indication that the data deviate systematically from the line of identity as is to be expected if the permeability of water at the BBB is limited. At low flows, there is, moreover, a slight tendency of the ASL-*K*_1_values to overestimate the MS-derived perfusion.

The latter effect can easily be explained by observing that we had to fix two quantities, namely *V*_*d*_ and *ρ*_*a*_ in Equation 8 in order to derive *K*_1_. These values are not known precisely, and this uncertainty does introduce potential bias in the *K*_1_estimate. We account for this bias by modifying the standard Renkin-Crone formula through multiplication with a scale factor *N* whose adjustment in the fit can compensate for this bias. The resulting value, *N*=(1.25±0.17), can be interpreted as an indication that the chosen value of the product *V*_*d*_·*ρ*_*a*_ is too high by about 25±17% which seems perfectly possible: reducing both *V*_*d*_and *ρ*_*a*_ by about 10% would suffice to eliminate the observed bias.

A similar *K*_1_(*f*) dependency (*K*_1_slightly overestimating blood flow at low flow rates and increasingly underestimating it beyond *f*=1 mL/min/mL) has been also reported by Parkes and Tofts who used single compartment fits to simulated signal curves generated with a two-compartment model proposed by these authors (see Figure 4a in [31]). They, moreover, report a field strength dependency of the deviations from the line of identity which should decrease at decreasing field strength. All deviations, including the overestimate at low flows, are interpreted as a consequence of the one compartment model simplification. Although the simulations are not directly comparable to the present study (and the used one-compartment operational equation differs from ours), the qualitative agreement with our fitted *K*_1_(*f*) curve is remarkable.

Adopting our approach of including the additional model parameter *N* in the fit, we obtain *PS*=(1.53±0.46) mL/min/mL as our best estimate of the *PS*-product of water at the BBB of the rat. Although the statistical uncertainty of this result is large (30%), this result is comparable to the available information regarding this parameter.

In a PET study, Herscovitch and Raichle [32] reported a value of *PS*=(1.04±32) mL/g/min for water at the BBB of rhesus monkeys. Takagi and coworkers [33] performed ASL measurements at the rat brain and report 1.71 ± 0.86 mL/g/min. Parkes and Tofts [31] also referred to earlier publications where the values in whole human brain varied from 0.9 to 1.7 min^−1^ with a mean value of 1.2 min^−1^[34,35]. We, therefore, consider the consistency of our result with the previously reported figures as indication that our description of the water kinetics with diffusion limited one-compartment model is adequate.

Consequently, we consider our results as proof that the ASL-FAIR technique is suitable to quantify brain perfusion in the rat at low, normal, and moderately increased flows (up to about 2 mL/min/mL). At higher flows, the limited permeability leads to increasingly reduced first-pass extraction of water across the BBB, and the measured *K*_1_ becomes increasingly insensitive to further increases in blood flow. Below about 2 mL/min/mL, an extraction correction can be performed with the help of Equation 14 using the fitted values of *PS* and *N* (but note that *N* is directly proportional to the chosen value of *V*_*d*_·*ρ*_*a*_: if a different value were chosen for this product *N* would have to be adjusted accordingly). Although we consider our results as adequate proof of the quantitative capabilities of ASL-FAIR for perfusion measurements in the rat brain, it should be noted that our investigation has several obvious limitations.

The major concern which could be raised regards the fact that MS- and ASL-investigations might not probe the same physiologic state of the animal. MS-derived *f* reflects a snapshot of the blood flow level a few seconds after MS injection. ASL, on the other hand, required about 15 min measurement per slice (more than half an hour for both investigated slices) and was, moreover, performed sequentially with the MS-investigation (before or after MS, depending on radioactive label, see Figure 1). The ASL-measurements thus provide time-averages which are, moreover, measured at rather different time points than the MS-derived flow. Of course, much care was taken to ensure stable physiologic conditions as far as possible, but residual variability cannot be excluded. We presume that part of the scatter in Figure 5 can be attributed to this residual variability and does not reflect the inherent limit of achievable statistical accuracy either with MS or ASL. There is, however, no reason to expect a systematic change (increase or decrease) of the flow level between both measurements since stable physiological conditions were closely monitored in each animal. As a further check, we performed a separate MR experiment in a single (identically treated) animal, which underwent four repeated ASL measurements over a much longer time period (2 h, 20 min) which yielded a constant *K*_1_ value of 1.2 mL/min/mL to within 8% (data not shown) which provides an estimate of the actually occurring perfusion changes in the given experimental setup. Moreover, PET and MR were performed in different order depending on the radioactive MS label used in the respective experiment without causing visible differences between both tracers in Figure 5. This is further evidence that no systematic flow change takes place between PET and MR measurement and that no sizable bias is present in the analysed *f* vs. *K*_1_correlation.

A further potential limitation of our study is the fact that we only compared slice averages of the target parameters (*f*,*K*_1_). While the slice thickness was comparable in MS and ASL (1 mm vs. 3 mm), the actual resolution mismatch implies a certain limitation of spatial correspondence. This, too, will contribute somewhat to the scatter in Figure 5. A spatially resolved comparison within the imaging planes was not performed since the combined image quality of MS and ASL was considered insufficient for such a comparison; also, regional differences were visible, notably in the ASL measurements. We believe that restriction to spatially averaged data does not adversely affect our accuracy: the MS-derived perfusion is simply related linearly to tracer uptake, and the slice averaged MS-associated radioactivity corresponds to the actual average perfusion in that region. The situation is markedly different for ASL. Therefore, the ASL data were quantified on a per-voxel basis and only averaged afterwards in order to obtain the correctly averaged uptake parameter *K*_1_.

Another restriction is the fact that the investigated flow range is limited to perfusion values below 1.9 mL/min/mL and that data above 1.0 mL/min/mL are scarce while exhibiting large fluctuations. Consequently, the derived *PS* value is not very precise, and validity of the Renkin-Crone assumption cannot be proven unambiguously. It, thus, would be desirable to perform further experiments targeting especially the high flow range.

A final important issue concerns the models applied for quantification of *f* and *K*_1_. While quantification of the MS data is completely straightforward and mathematically simple, quantification of the ASL data requires selection among quite a number of different modeling approaches [21-24,36-38].

We essentially follow the approach described in [22] which leads to Equation 8. Since it is possible that the one-compartment Kety-Schmidt model leads to underestimation of perfusion at high flow values due to limited permeability of the BBB to water [9,39], we do not postulate that the resulting parameter *K*_1_is identical to tissue perfusion *f*, but allow for a Renkin-Crone type relation between *K*_1_ and *f*. The underlying assumption of this approach is that even in the presence of limited permeability, the kinetics is reasonably well described by a one-compartment model. We believe that this is reasonable at least as long as the *PS* product is higher than or comparable to the relevant flow levels. Treating the capillary as a separate identifiable kinetic compartment (see, e.g. [23,24]) is of course possible, and different approximations and limiting cases have been investigated [31]. Given the limited statistical accuracy of the experimental data there is, in our view, no realistic possibility of identifying *f* and *PS* (plus the vascular fraction) simultaneously in the data (this assessment is in accord with [16]). The only really visible effect of limited *PS* is a reduction of tissue uptake *K*_1_ relative to *f* at elevated flow levels.

By independently measuring *f* via the MS experiments, we were able to determine experimentally the relation between *f* and the effective *K*_1_. This relation, as presented in Figure 5, is reasonably described by a Renkin-Crone type formula (Equation 13 or 14) which supports the above conjecture that our one-compartment description is adequate. We note that the Renkin-Crone relation is not compatible with the two-compartment model which would yield a different expression for the flow dependent extraction (and a different numerical value for *PS*), namely *E*(*f*)=*PS*/(*f* + *PS*). We believe the Renkin-Crone model to be more reasonable since it accounts for the arterio-venous concentration gradient in the capillary (it can be considered as a simple variant of a distributed model) instead of making the rather strong assumption that the capillary reacts as a well-mixed compartment (which obviously is not really the case). The statistical accuracy of our data, however, is insufficient to decide between the two alternative expressions for the function *E*(*f*). Still, this question is irrelevant as long as the objective of the experiment is not to determine the *PS* product itself. We take the point of view that the one-compartment model combined with the Renkin-Crone formula regarding the flow dependence of the unidirectional uptake rate *K*_1_ of the compartment model is phenomenologically sufficient for description of the data and for derivation of quantitative perfusion values from the ASL measurements.

## Conclusions

The ASL-FAIR technique is suitable for absolute blood flow quantification in the rat brain when using a one-compartment model including a suitable extraction correction for data evaluation.

## Competing interests

The authors declare that they have no competing interests.

## Authors’ contributions

AB performed the data analysis, is the main author of the manuscript, and carried out experiments together with RB. KS initially tested the sequence used for ASL measurements. FH performed part of the data analysis. JS provided intellectual input. JvdH formulated the modeling part of this work and supported the discussion with his experience and knowledge of the subject. All authors reviewed the manuscript. All authors read and approved the final manuscript.
